# Public health risks related to food safety issues in the food market: a systematic literature review

**DOI:** 10.1186/s12199-019-0825-5

**Published:** 2019-11-30

**Authors:** Zemichael Gizaw

**Affiliations:** Department of Environmental and Occupational Health and Safety, Institute of Public Health, College of Medicine and Health Sciences, University of Gondar, Gondar, Ethiopia

**Keywords:** Public health risks, Public health hazards, Public health problems, Food safety, Food quality, Food hygiene, Food marketing

## Abstract

**Background:**

Food safety in the food market is one of the key areas of focus in public health, because it affects people of every age, race, gender, and income level around the world. The local and international food marketing continues to have significant impacts on food safety and health of the public. Food supply chains now cross multiple national borders which increase the internationalization of health risks. This systematic review of literature was, therefore, conducted to identify common public health risks related to food safety issues in the food market.

**Methods:**

All published and unpublished quantitative, qualitative, and mixed method studies were searched from electronic databases using a three step searching. Analytical framework was developed using the PICo (population, phenomena of interest, and context) method. The methodological quality of the included studies was assessed using mixed methods appraisal tool (MMAT) version 2018. The included full-text articles were qualitatively analyzed using emergent thematic analysis approach to identify key concepts and coded them into related non-mutually exclusive themes. We then synthesized each theme by comparing the discussion and conclusion of the included articles. Emergent themes were identified based on meticulous and systematic reading. Coding and interpreting the data were refined during analysis.

**Results:**

The analysis of 81 full-text articles resulted in seven common public health risks related with food safety in the food market. Microbial contamination of foods, chemical contamination of foods, food adulteration, misuse of food additives, mislabeling, genetically modified foods (GM foods), and outdated foods or foods past their use-by dates were the identified food safety–related public health risks in the food market.

**Conclusion:**

This systematic literature review identified common food safety–related public health risks in the food market. The results imply that the local and international food marketing continues to have significant impacts on health of the public. The food market increases internationalization of health risks as the food supply chains cross multiple national borders. Therefore, effective national risk-based food control systems are essential to protect the health and safety of the public. Countries need also assure the safety and quality of their foods entering international trade and ensure that imported foods conform to national requirements.

## Background

Food safety is an important issue that affects all of the world’s people. Many countries throughout the world are increasingly interdependent on the availability of their food supply and on its safety. Hence, people all over the world increasingly value food safety; food production should be done safely to maximize public health gains and environmental benefits. Food safety deals with safeguarding the food supply chain from the introduction, growth, or survival of hazardous microbial and chemical agents [1, 2].

Unsafe food containing harmful bacteria, viruses, parasites, or chemical substances causes more than 200 diseases—ranging from diarrhea to cancers. An estimated 600 million in the world fall ill after eating contaminated food and 420,000 die every year, resulting in the loss of 33 million disability adjusted life years (DALYs). Children under 5 years of age carry 40% of the food borne disease burden, with 125,000 deaths every year. Diarrheal diseases are the most common illnesses resulting from the consumption of contaminated food, causing 550 million people to fall ill and 230,000 deaths every year [3].

Food safety is being challenged nowadays by the global dimensions of food supply chains [1, 4, 5]. Foods in the international market may be frauded as different parties such as manufacturers, co-packers, distributors, and others along the chain of distribution involve in the national or international trade [6–8]. Food safety in the food market is one of the key areas of focus in public health, because it affects people of every age, race, gender, and income level around the world. The local and international food marketing continues to have significant impacts on food safety and health of the public. Food supply chains now cross multiple national borders which increase the internationalization of health risks [9–14]. This systematic review of literature was, therefore, conducted to identify common public health risks related to food safety issues in the food market. This review provides evidence to improve food safety in the food market using risk-based food safety strategies. Healthcare providers, researchers, and policy makers may use the results of this systematic literature review to protect the public from undue health effects due to consumption of foods with poor quality and safety.

## Methods

### Research question

What food safety–related public health risks are commonly found in the food market?

### Analytical framework

We developed the components of the analytical framework using the PICo (population, phenomena of interest, and context) method. The population for this review was the public over the globe. The phenomenon of interest for this review was public health risks associated with food safety. The context was the food market (such as restaurants, food stores, supermarkets, shops, food processing plants, and street vending). The reviewers sat together to discuss and refine the framework.

### Criteria for considering studies for this review

All published and unpublished quantitative, qualitative, and mixed method studies conducted on food safety–related health risks for the general public in the food market were included. Governmental and other organizational reports were also included. Articles published other than English language, citations with no abstracts and/or full texts, duplicate studies, and studies with poor quality were excluded.

### Search strategy

We searched published articles/or reports from MEDLINE/ PubMed, EMBASE, CINAHL, Access Medicine, Scopus, Web of Science, Google Scholar, WHO Library, FAO Libraries, and WTO Library. We also searched thesis and dissertations from Worldcat and ProQuest. We used a three step searching. In the first step, we conducted an initial limited search of MEDLINE and analyzed the text words contained in the title and abstract, and of the index terms used to describe articles. Secondly, we searched across all included databases using all identified keywords and index terms. Thirdly, references of all identified articles were searched to get additional studies. The search term we used in the initial searching is presented as follows.

((((("public health"[MeSH Terms] OR ("public"[All Fields] AND "health"[All Fields]) OR "public health"[All Fields]) AND ("risk"[MeSH Terms] OR "risk"[All Fields] OR "risks"[All Fields])) OR (("public health"[MeSH Terms] OR ("public"[All Fields] AND "health"[All Fields]) OR "public health"[All Fields]) AND hazards[All Fields])) OR (("public health"[MeSH Terms] OR ("public"[All Fields] AND "health"[All Fields]) OR "public health"[All Fields]) AND problems[All Fields])) AND ((("food safety"[MeSH Terms] OR ("food"[All Fields] AND "safety"[All Fields]) OR "food safety"[All Fields]) OR ("food quality"[MeSH Terms] OR ("food"[All Fields] AND "quality"[All Fields]) OR "food quality"[All Fields])) OR (("food"[MeSH Terms] OR "food"[All Fields]) AND ("hygiene"[MeSH Terms] OR "hygiene"[All Fields])))) AND (((("food"[MeSH Terms] OR "food"[All Fields]) AND market[All Fields]) OR (("food"[MeSH Terms] OR "food"[All Fields]) AND trade[All Fields])) OR (("food supply"[MeSH Terms] OR ("food"[All Fields] AND "supply"[All Fields]) OR "food supply"[All Fields]) AND chain[All Fields]))

### Assessment of methodological quality

Search results from different electronic databases were exported to Endnote reference manager to remove duplication. Two independent reviewers (ZG and BA) screened out articles using titles and abstracts. The reviewers further investigated and assessed full-text articles against the inclusion and exclusion criteria. The reviewers sat together to resolve disagreements during the review. The methodological quality of the included studies was assessed using mixed methods appraisal tool (MMAT) version 2018 [15]. This method explains the detail of each criterion. The rating of each criterion was, therefore, done as per the detail explanations included in the method. Almost all of the included full-text articles fulfilled the criteria and all the included full-text articles were found to be better quality.

### Data extraction

In order to minimize bias, we the reviewers independently extracted data from papers included in the review using JBI mixed methods data extraction form [16]. The data extraction form was piloted on randomly selected papers and modified accordingly. Eligibility assessment was performed independently by the two reviewers. Information like authors, year of publication, study areas, type of studies, and focus of the study or main messages were extracted.

### Synthesis of findings

The included full-text articles were qualitatively analyzed using emergent thematic analysis approach to identify key concepts and coded them into related non-mutually exclusive themes. We then synthesized each theme by comparing the discussion and conclusion of the included articles. Emergent themes were identified based on meticulous and systematic reading. Coding and interpreting the data were refined during the analysis.

## Results

### The search process

**Fig. 1:**
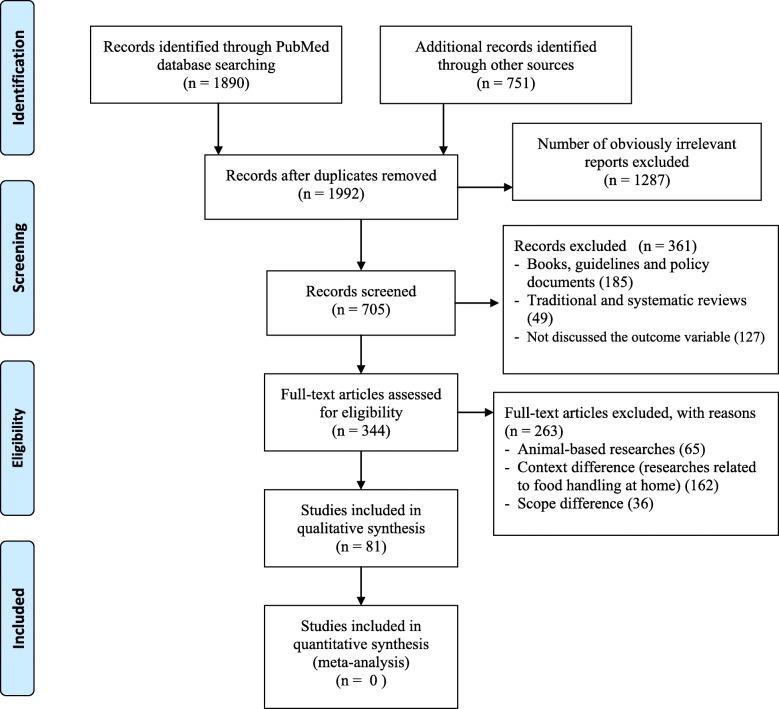
Study selection flow diagram

The search strategy identified 2641 titles and abstracts (1890 from PubMed and 751 from other sources) as of 13 June 2019. We obtained 1992 title and abstracts after we removed duplicates. Following assessment by title and abstract, 705 articles were retrieved for more evaluation and 344 articles were assessed for eligibility. Finally, 81 articles were included for systematic literature review based on the inclusion criteria (Fig. 1).

**Fig. 2:**
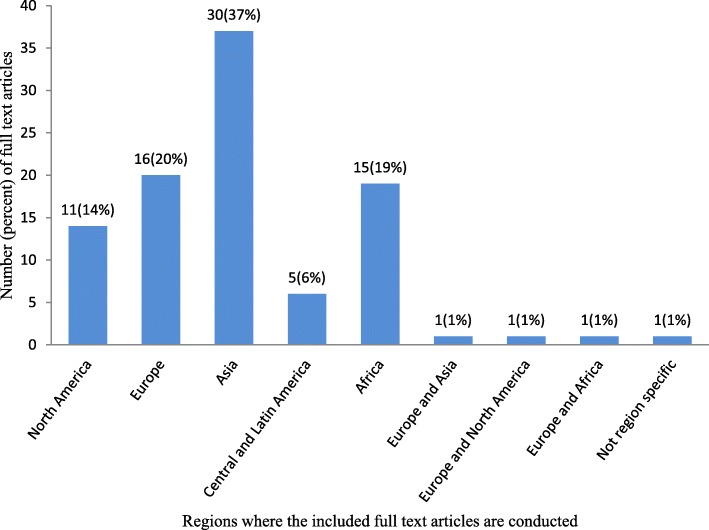
Regions where the included full-text articles conducted

In this review, 81 of 1992 (4%) full-text articles matched the inclusion criteria. The overwhelming majority, 74 of 81 (91%) of the included full-text articles are research articles; 2 (3%) are short communications; 2 (3%) are regulatory papers, 1 (1%) is field inspection; 1 (1%) is research note; and the other 1 (1%) is thesis. Of the included full-text articles, 30 of 81 (37%) are conducted in Asia; 4 of 81 (5%) are conducted in multiple countries in the same region or across regions; and 1of 81 (1%) is not region specific (Fig. 2).

All the included full-text articles are published between 1991 and 2018 (35 (43%) between 2011 and 2015; 16 (20%) between 2000 and 2005; 16 (20%) between 2006 and 2010; 12 (15%) between 2016 and 2018; and the rest 2(2%) before 2000).

### Food safety–related public health risks identified from the search process

The analysis of 81 full-text articles resulted in seven common public health risks related with food safety in the food market. Microbial contamination of foods, chemical contamination of foods, food adulteration, misuse of food additives, mislabeling, GM foods, and foods past their use-by dates were the identified food safety–related health risks in the food market (Table 1).

**Table 1:** Common food safety–related public health risks identified from the search process

Common public health risks related with food safety	Number of papers
Microbial contamination of foods	21
Chemical contamination of foods	15
Food adulteration	9
Misuse of food additives	11
Mislabeling	17
Genetically modified foods	4
Foods past their use-by dates	6

Table 2 shows food safety–related public health risks in the food market by country name (countries are categorized into developed and developing based on the United Nations (UN) 2019 list). Among 21 full-text articles included for microbial contamination of foods, 13 (62%) were from developing countries. This may suggest microbial contamination of foods in the food market is a common public health risk in developing countries than the developed. Eight (53%) of 15 articles retrieved for chemical contamination of foods in the food market were from developing countries. The vast majority, 8 of 9 (89%) full-text articles retrieved for food adulteration were from developing countries, which may indicate adulteration of foods is practiced more of in developing countries. Similarly, 8 of 11 (73%) of the full-text articles included for misuse of food additives were from developing countries, which may show misuse of food additives is a common problem in developing countries. For mislabeling, 14 of 17 (82%) and 8 of 17 (47%) of the full-text articles were from developed and developing countries respectively. Four out of six (67%) of full-text articles retrieved for foods past use-by dates were from developing countries. This may show selling of outdated foods is common in developing countries than the developed.

**Table 2:** Food safety–related public health risks in the food market by country name (countries are categorized into developed and developing based on the United Nations (UN) 2019 list)

Food safety issues	Countries where the included full-text articles are conducted	
	Developed	Developing
Microbial contamination (21)	Italy	Philippines
	USA	Nigeria (2)
	Greece (2)	Mexico (2)
	Spain (2)	Sudan
	UK (2)	India (2)
		South Africa
		Iran
		Thailand
		Tanzania
		Bangladesh
Chemical contamination (15)	Saudi Arabia (2)	China (2)
	USA	Tunisia
	Belgium (2)	Nigeria (2)
	Canada	Egypt
	Italy	Bangladesh (2)
Food adulteration (9)	Taiwan	Bangladesh (3)
		India (2)
		Pakistan
		Ethiopia (2)
Misuse of additives (11)	USA (2)	India (4)
	Taiwan	Pakistan(2)
		Iran
		Indonesia
Mislabeling (17)^a^	Ireland	China
	USA (3)	Malaysia
	Italy (3)	India
	Spain (4)	Egypt (2)
	Greek	South Africa
	Canada	Brazil (2)
	Belgium	
Genetically modified foods (4)^b^	USA	Eastern Caribbean
	Canada	
Food past use-by dates (6)	Canada	Nigeria
	USA	Indonesia
		Bangladesh
		Kenya

Numbers in the bracket show the number of full-text articles included

^a^There are studies conducted in two and/or three different countries. In this case, we may count one study twice and /or three times.

^b^One study was conducted in a general context. So, we did not include it when we categorize studies in regions

**Fig. 3:**
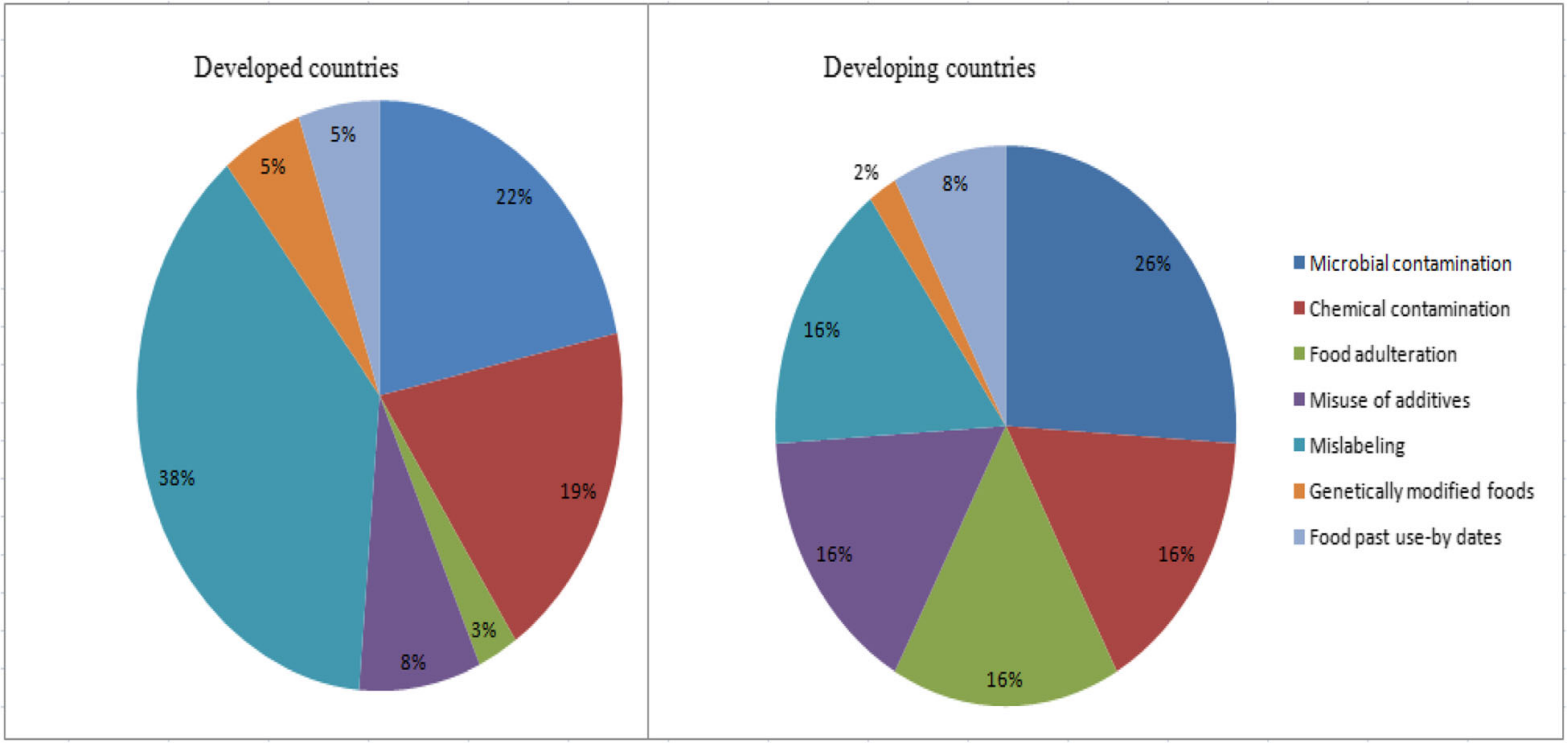
Comparison of food safety issues in developed and developing countries

Figure 3 shows comparison of food safety issues in developed and developing countries. A total of 37 and 50 articles were included in this review from developed and developing countries respectively. The comparison of food safety issues among developed countries suggests that mislabeling (38%), microbial contaminations (22%), and chemical contamination (19%) are the commonest food safety issues in the food market. Similarly, the comparison of food safety issues among developing countries suggests that microbial contaminations (26%), chemical contaminations (16%), food adulteration (16%), misuse of additives (16%), and mislabeling (16%) are the commonest food safety issues in the food market.

### Microbial contamination of foods

In this review, 21 of 81 (26%) full-text articles reported the presence of pathogenic microorganisms in different food items in the food market. These studies identified different diseases causing bacteria mainly *Salmonella* spp., *Escherichia coli*, *Klebsiella* spp., *Shigella* spp., *Enterobacter* spp., *Proteus* spp., *Citrobacter* spp. *Staphylococcus aureus*, Campylobacter spp., *Listeria* spp., *Vibrio*, *Alklegens* spp., *Bacillus cereus*, Pseudomonas spp., *Clostridium perfringens*, Arcobacter spp., and *Enterococcus* spp. Moreover, different fungus such as Blastomyces, *Fusarium* spp., *Mucor* spp., *Aspergillus niger*, *Fusarium avenaceum*, *Penicillium digitatum*, *Rhizopus stolonifer*, *Saccharomyces* species, *Fusarium solani*, *Aspergillus flavus*, *Saccharomyces dairensis*, and *Saccharomyces exiguus* were identified from different food items from food stores or shops. The included studies also reported that some of the microorganisms are resistant to different antimicrobials (Table 3). The results also show that total coliforms, fecal coliforms, and different fungus were commonly reported in developing countries than developed countries. On the other hand, different Campylobacter species were reported in developed countries than developed countries.

**Table 3:** Summary of full-text articles which reported microbial contamination of foods as a public risk in food marketing

Authors	Country/region	Article type	Main message/findings
Gabriel AA, et al., 2007 [17]	Philippines	Research article	This study assessed the microbiological quality of retailed mung bean sprouts. Ninety-four percent of the samples tested positive for the presence of *Salmonella* spp. and some samples had Coliform and *Escherichia coli* counts as high as 5.90 and 5.50 log_10_ CFU g^−1^, respectively. The poor microbiological quality of most of the tested sprouts was attributed to unhygienic sprout production and retailing practices.
Adeyanju GT and Ishola O, 2014 [18]	Nigeria	Research article	Accessed the levels of *Salmonella* and *Escherichia coli* in frozen poultry meats including their antimicrobial resistance pattern in Ibadan. Thirty-three percent and 43.4% of samples from retail markets tested positive for *Salmonella* and *Escherichia coli* respectively. *Salmonella enterica* spp. showed 93% resistance to tetracycline and 100% resistance to augmentin and amoxicillin, while *Escherichia coli* showed 100% resistance to augmentin and amoxicillin.
Giammanco GM, et al., 2011 [19]	Italy	Research article	This study assessed common food pathogens in cheese collected from retailing markets in Palermo. The result indicated that 4% and 44% of the samples, respectively, did not comply with the acceptability levels for *S. aureus* and *E. coli*. A high contamination of bacteria belonging to *Enterobacteriaceae* and *Staphylococcaceae* was found in 42% and 50% of the cheeses analyzed, respectively. The results indicated that poor husbandry and poor hygiene practices during milk collection or preservation or during cheese production processes and handling. In addition, the retail sale conditions may have played a role in cheese contamination.
Zhao C, et al., 2001 [20]	USA	Research article	This study assessed the prevalence of common food pathogens from retail raw meats in Washington, DC. Results of the study showed that 70.7% of chicken samples were contaminated with *Campylobacter*. Approximately 14% of turkey samples yielded *Campylobacter*, whereas fewer pork (1.7%) and beef (0.5%) samples were positive for *Campylobacter*. Thirty-eight point seven percent of chicken samples yielded *E. coli*, while 19.0% of the beef samples, 16.3% of the pork samples, and 11.9% of the turkey samples were positive for *E. coli*. However, only 3.0% of the retail meat samples tested were positive for *Salmonella*.
Cárdenas C, et al., 2013 [21]	Mexico	Research article	This study evaluated the microbiological quality of tomatoes and peppers from markets and supermarkets in Monterrey, Mexico. The results showed that the presence of indicator organisms was relatively high in peppers (average 4.4 to 4.7 log CFU/g for total mesophilic, 3.25 to 3.73 log CFU/g for total coliforms, and 1.69 log CFU/g for fecal coliforms). Tomatoes and peppers showed the greatest microorganism levels (~ 1 log CFU/g higher) in comparison with the other varieties.
Filiousis G, et al., 2009 [22]	Greece	Short communication	This study analyzed prevalence, genetic diversity, and antimicrobial susceptibility of *Listeria monocytogenes* isolated from open-air food markets in Thessaloniki, Greece. Thirty (14.3%) contained *L. monocytogenes* with the highest prevalence in raw meat (27.5%), raw meat products (18%), and cheese (8%). The strains were susceptible to 16 antimicrobials, except one strain which displayed resistance to tetracycline.
Pérez-Rodríguez F, et al., 2010 [23]	Spain	Research article	This study evaluated hygiene practices and microbiological quality of cooked meat products during slicing and handling at retail in Cordoba, Spain. *Listeria monocytogenes* and *Listeria inocua* were isolated from 7.35% (5/68) and 8.82% (6/68) of analyzed samples, respectively. Deficient handling practices were more common in small sized establishments.
Yagoub SO, 2009 [24]	Sudan	Research article	This study aimed to isolate *Enterobacteriaceae* and *Pseudomonas* spp. from raw fish sold in fish market in Khartoum. *Enterobacteriaceae* were isolated from 83 out of 150 (55%) randomly collected fishes, the most dominant isolates were *E. coli*, *Citrobacter* spp., *Enterobacter* spp., and *Klebsiella* spp. This together with the highly pathogenic *Enterobacteriaceae* including *Salmonella* spp. and *Shigella* spp., *Proteus* spp., and *Alklegens* spp. Potential pathogenic organisms were also among the isolates. On the other hand, *Pseudomonas* spp. were isolated from 62% of randomly collected fishes.
Kumari S and Sarkar PK, 2014 [25]	India	Research article	This study characterized *Bacillus cereus* group from various marketed dairy products in India. The prevalence of *B. cereus* group in cheese, ice cream, milk powder, and milk was high (33–55%), whereas it was low in butter and paneer samples (20% and 4%, respectively). The level of contamination in the various dairy products was up to 10^8^ cfu g^−1^ or ml^−1^. An antibiogram of 144 isolates of *B. cereus* group was obtained using 14 different antibiotics commonly used against foodborne diseases. All the 144 isolates were multidrug (at least five antibiotics) resistant.
Domınguez C, et al., 2002 [26]	Spain	Research article	This study assessed prevalence of *Salmonella* and *Campylobacter* in retail chicken meat in Spain. *Salmonella* was isolated from 71 (35.83%) of the samples analyzed. The predominant serovars were *S. enteritidis* (47.88%), *S. hadar* (25.35%), and serotype 4,12:b:-(II) (19.71%). Other serovars such as *S. mbandaka*, *S. derby*, *S. virchow*, and *S. paratyphi B* were isolated in much lower levels. Thermophilic campylobacters were isolated in 49.50% of the samples studied.
Vantarakis A, et al., 2011 [27]	Greece	Research article	This study assessed occurrence of microorganisms of public health and spoilage significance in fruit juices sold in retail markets in Greece. Bacteria were isolated from 51 samples (42.5%) and fungi from 78 samples (65%). *Escherichia coli O157:H7* was detected in four of the analyzed samples (3.34%), and *Staphylococcus aureus* was detected in four different samples (3.34%). In 11 samples (9.1%), the total number of microorganisms detected was as high as 125 CFU. Acidophilic microorganisms were isolated from 26 samples (21.7%) and *Blastomyces* was detected in 46 samples (38.3%).
Heredia N, et al., 2001 [28]	Mexico	Research note	This study assessed microbiological Condition of Ground Meat Retailed in Monterrey, Mexico. Over 75% of the samples contained 10^5^ total mesophilic microorganisms per g, and over 40% had 10^6^ total coliforms per g. Fecal coliforms were present in most samples. *Staphylococcus aureus* was detected in 2.3% of the samples, *Salmonella* spp. in 11.4%, *Listeria* spp. in 62%, and *L. monocytogenes* in 16%. *Escherichia coli* was detected in 76% of samples. *Fusarium* spp. and *Mucor* spp. were detected in 3.4% of the samples, and low levels of yeast in 93%.
Nel S, et al., 2004 [28]	South Africa	Research article	This study assessed bacterial populations associated with meat from the deboning room of a high-throughput red meat abattoir in South Africa. Almost the counts exceeded the microbiological guidelines for raw meat. The average *B. cereus* count over the sampling period was 8.32 × 10^3^ cfu, g^−1^, for *S. aureus* and *Pseudomonas* spp. 1.72 × 10^5^ and 1.7 × 10^5^ cfu g^−1^ respectively and for *E. coli* 3.4 × 10^5^ cfu g^−1^. Sixty percent of the samples were positive for presumptive *Salmonella* spp. while 52% of the samples tested positive for the presence of *L. monocytogenes*. The aerobic plate and Enterobacteriaceae counts were 1.7 × 10^7^and 4.6 × 10^6^ cfu g^−1^, respectively.
Elson R, et al., 2004 [29]	UK	Research article	This study examined microbiological quality of ready-to-eat cold sliced meats from catering and retail premises in the UK. Most ready-to-eat meat samples (75%) were of satisfactory/acceptable microbiological quality and 25% were of unsatisfactory/unacceptable quality. Two cold meat samples (< 1%) were of unacceptable microbiological quality because of the presence of *Campylobacter jejuni* in 25 g and *Listeria monocytogenes* at 3.4 × 10^4^ CFU g^−1^.
Hosseini A. 2011 [30]	Iran	Research article	This study examined bacterial contamination of table eggs from retails markets in Iran. The result showed that 19 samples were contaminated by *E. coli*, four samples by *Proteus* spp., and one sample by *Klebsiella* spp*.* Average colony count of coli form bacteria was 20 cfu/g and *E. coli* was 12/6 cfu/g.
Banerjee M and Sarkar PK, 2003 [31]	India	Research article	This study investigated microbiological quality of some retail spices in India. The total aerobic mesophilic bacteria count showed that 51% of the samples were in the unacceptable level (> 10^6^ cfu g^−1^). While molds were detected in 97% of the samples, yeast was found in only one. *Bacillus cereus*, *Clostridium perfringens*, *Staphylococcus aureus*, and members of Enterobacteriaceae occurred in 85, 59, 11, and 85%, respectively of the kinds. Coliforms and fecal coliforms were found in 33 and 15%, respectively of the kinds. *Escherichia coli* was detected in only one sample, of garlic. *Salmonella* and *Shigella* were found only in 2.6% of the samples.
Vindigni SM, et al., 2007 [32]	Thailand	Research article	This study assessed prevalence of foodborne microorganisms in retail foods in Thailand. Of the 200 samples tested, 121 (61%) were positive for at least one *Salmonella* spp. serogroup. A total of 175 *Salmonella* spp. were isolated. The most common serotype was *Salmonella* Anatum, followed by S. Corvallis and S. Derby. *Campylobacter* spp. were found in 31 (15.5%) of 200 samples. *C. jejuni* was isolated from 15% of fresh market chicken samples and 35% of supermarket chicken samples. *Arcobacter* spp. were isolated from 42 (21%) samples; fresh market chicken had significantly higher *A. butzleri* contamination than supermarket chicken. The presence of *Enterococcus* spp., an indication of fecal contamination, was detected in 188 (94%) samples, including 100% of the beef and pork sources.
Simforian E, et al., 2015 [33]	Tanzania	Research article	This study assessed microbiological quality of raw fruit juice in Tanzania. The results showed that the total plate counts (TPC) ranged between 2.32 and 8.54 (Log cfu/ml). About 72.2% of juice samples had TPC above Codex recommended maximum levels (3.7–4.7 Log cfu/ml). The prevalence of *Escherichia coli* in the juices was 80% with a range between 0.0 and 5.0 (Log MPN/ml) suggesting of direct fecal contamination or contamination from the environment.
Mailafia S, et al., 2017 [34]	Nigeria	Research article	This study identified fungi associated with spoilt fruits vended in Gwagwalada market. Nigeria *Aspergillus niger* had the highest occurrence in pineapple, watermelon, oranges, pawpaw, and tomatoes with a frequency of 38%. *Fusarium avenaceum* followed with the frequency of occurrence of 31% in fruits such as pineapple, watermelon, oranges, pawpaw, and tomatoes while *Penicillium digitatum* and *Rhizopus stolonifer* had the least frequency of 4% each in tomato; and orange and tomato, respectively. Other fungal species were identified as yeast (*Saccharomyces* species) (10%), *Fusarium solani* (8%), and *Aspergillus flavus* (5%). The highest prevalence rate was 70% of *A. niger* from orange followed by *F. avenaceum* of which 65% isolates were recovered from pawpaw. Other fungal organisms such as yeast (*Saccharomyces* species), *P. digitatum*, and *R. stolonifer* were isolated with varying prevalence (40%, 20%, and 5%) from watermelon, tomato, and orange, respectively.
Hunter PR, et al., 1994 [35]	England	Research article	This study isolated food spoilage yeasts from salads purchased from delicatessens in the Warrington area, England. The results indicated that Of the 87 salads, only 19% had plate counts greater than 10,000 organisms/g. Coliforms were isolated from 3 samples, *E. coli* from one, and *Listeria monocytogenes* from one. By contrast, yeasts were isolated from 76% of the salads and at counts greater than 10,000 organisms/g in 31%. Twenty-one different yeast species were isolated, of which the most common were *Saccharomyces dairensis* and *Saccharomyces exiguus*.
Islam M, 2017 [36]	Bangladesh	Thesis	This study assessed bacteriological quality of street-vended and expired food items collected from different areas in Dhaka City, Bangladesh. Out of total 35food samples (expired and street), enteric bacteria were found in 17 (48.6%) food samples containing *E. coli*, *Vibrio*, *Shigella*, and *Salmonella* species.

### Chemical contamination of foods

Fifteen (19%) of the full-text articles included in this review reported that contamination of foods with hazardous chemicals is a major public health concern associated with the food market. Heavy metals (like cadmium, nickel, lead, copper, zinc, iron, mercury, and manganese), pesticide residuals (like dichlorvos, dimethoate, parathion-methyl, pirimiphos-methyl, and parathion), persistent organic pollutants (like dichlorodiphenyltrichloroethane metabolites, polychlorinated biphenyls, perfluorooctanoic acid, endosulfans, and aldrin), organic compounds (like patulin, chloroform, formalin, and urea), volatile organic compounds (like ethyl benzene, o-xylene, and benzene), hydrocarbons (like benzo[a]pyrene and toluene), and other chemical compounds (like calcium carbide and cyanide) are chemical contaminants identified by the full-text articles included in this review. In most cases, the concentration of chemicals exceeded the tolerable limit for consumable food items (Table 4).

**Table 4:** Summary of full-text articles which reported chemical contamination of foods as a public risk in food marketing

Authors	Country/region	Article type	Main message/findings
Bai Y, et al., 2006 [37]	China	Research article	This study investigated the organophosphorus (OP) pesticide residues in market foods in China. In 18 of 200 samples, five OP pesticides, including dichlorvos, dimethoate, parathion-methyl, pirimiphos-methyl, and parathion, were found in concentrations ranging from 0.004 to 0.257 mg/kg. The mean levels of dimethoate in fruits and parathion in vegetables exceeded the maximum residue limits (MRLs).
Othman ZAA, 2010 [38]	Saudi Arabia	Research article	This study determined lead contamination in the Riyadh market in Saudi Arabians. Results showed that sweets (0.011–0.199 μg/g), vegetables (0.002–0.195 μg/g), legumes (0.014–0.094 μg/g), eggs (0.079 μg/g), and meat and meat products (0.013–0.068 μg/g) were the richest sources of lead.
Zaied C, et al., 2013 [39]	Tunisia	Research article	This study assessed occurrence of patulin in apple-based foods from supermarkets and stores in Tunisia. Results showed that the incidence of patulin contamination was 35%. The levels of contamination determined in the total samples ranged between 0 and 167 mg/l with a mean value of 20 mg/l and a median of 13 mg/l. Eighteen percent (18%) of the total juice samples (apple juices and mixed juices) and twenty-eight percent (28%) of the baby food samples exceeded the tolerable limit recommended by the European Union, which are respectively 50 mg/l and 10 mg/l.
Schecter A, et al., 2010 [40]	USA	Research article	This study assessed contamination of foods by persistent organic pollutants (POPs) in the USA. Results showed that the highest level of pesticide contamination was from the dichlorodiphenyltrichloroethane (DDT) metabolite *p*,*p*´dichlorodiphenyldichloroethylene, which ranged from 0.028 ng/g wet weight (ww) in whole milk yogurt to 2.3 ng/g ww in catfish fillets. Authors found polychlorinated biphenyls (PCB) congeners (28, 52, 101, 118, 138, 153, and 180) primarily in fish, with highest levels in salmon (PCB153, 1.2 ng/g ww; PCB138, 0.93 ng/g ww). For PFCs, we detected perfluorooctanoic acid (PFOA) in 17 of 31 samples, ranging from 0.07 ng/g in potatoes to 1.80 ng/g in olive oil. In terms of dietary intake, DDT and DDT metabolites, endosulfans, aldrin, PCBs, and PFOA were consumed at the highest levels.
Onianwa P, et al., 2001 [41]	Nigeria	Research article	This study determined concentrations of copper and zinc in food items of various classes which were obtained from the markets of Nigeria. The results showed that copper levels ranged widely from 0.06 to 13.3 mg/kg, while zinc levels ranged from 0.06 to 56.9 mg/kg in various foods. Highest levels of both metals were found to occur in legumes (Cu, 8.3 ± 3.7 mg/kg; Zn, 29 ± 12 mg/kg). The estimated weighted average dietary intakes for the entire adult population were calculated to be 2.64 mg Cu/day and 15.8 mg Zn/day.
Vinci RM, et al., 2015 [42]	Belgium	Research article	This study assessed occurrence of volatile organic compounds (VOCs) in foods from the Belgian market. The results showed that the most prevalent OVCs and respective percentages of occurrence were as follows: chloroform (97%), toluene (95%), ethyl benzene (80%), o-xylene (79%), and benzene (58%). The maximum probabilistic dietary intake was with 0.151, 0.645, 0.138, 0.066, and 0.118 mg kg bw1 day1 for chloroform, toluene, ethyl benzene, o-xylene, and benzene respectively.
Tittlemier SA, et al., 2004 [43]	Canada	Research article	This study assessed polybrominated diphenyl ether (PBDE) in retail fish and shellfish samples purchased from Canadian markets. The results showed that trout and salmon contain1600 and 1500 pg/g, wet weight, respectively. The concentration of PBDE was found to be 260, 180, and 48 pg/g, wet weight, respectively in mussel, tilapia, and shrimp.
Onianwa P, et al., 2000 [41]	Nigeria	Short communication	This study determined cadmium and nickel composition of Nigerian foods. The results indicated that cadmium levels ranged from 0.01 to 0.62 mg/kg, with a general average of 0.16 ± 0.14 mg/kg. Cadmium levels varied significantly between different groups of foods, with the highest levels occurring in dairy (0.41 ± 0.25 mg/kg), and the lowest in confectioneries and fruits (0.07 ± 0.04 mg/kg). Nickel levels ranged from 0.05 to 9.22 mg/kg with a general average of 2.1 ± 1.5 mg/kg. The levels of both metals were found to be higher than the levels observed in similar foods in some developed countries.
Radwan MA and Salama AK, 2006 [44]	Egypt	Research article	This study assessed the level of heavy metals in Egyptian fruits and vegetables The results of this survey showed that the average concentrations detected were ranged from 0.01 to 0.87, 0.01 to 0.15, 0.83 to 18.3, and 1.36 to 20.9 mg/kg for Pb, Cd, Cu, and Zn, respectively. The highest mean levels of Pb, Cd, Cu, and Zn were detected in strawberries, cucumber, date, and spinach, respectively.
Ali MH and Al-Qahtani KM, 2012 [45]	Saudi Arabia	Research article	This study assessed concentration of heavy metals in vegetables, cereals, and fruits in Saudi Arabian markets. The results declared that concentrations of major studied metals were exceeding than the recommended maximum acceptable levels proposed by the Joint FAO/WHO Expert Committee. Leafy vegetables were found to contain the highest metal values especially parsley (543.2 and 0.048 μg/g for Fe and Hg respectively), Jews mallow (94.12 and 33.22 μg/g for Mn and Zn respectively), spinach (4.13 μg/g for Cd). While peas in legumes group maintained the highest Zn content 71.77 μg/g and finally cucumber had the highest Pb content 6.98 μg/g on dry matter basis.
NIE Ji-yun, et al., 2016	China	Research article	This study analyzed the concentrations of the heavy in China’s main deciduous fruits. Only 2.2% of the samples were polluted by Ni, only 0.4% of the samples were polluted by Pb, and no samples were polluted by Cd or Cr. For the combined heavy metal pollution, 96.9% of the samples were at safe level, 2.32% at warning level, 0.65% at light level, and 0.13% at moderate level.
Vinci RM, et al., 2012 [42]	Belgium	Research article	This study assessed human exposure to benzene through foods from the Belgian market. Benzene was found above the level of detection in 58% of analyzed samples with the highest contents found in processed foods such as smoked and canned fish, and foods which contained these as ingredients (up to 76.21 μg kg^−1^).
Moret S, et al., 2010 [46]	Italy	Research article	This study assessed levels of polycyclic aromatic hydrocarbons (PAHs) in dietary supplements from the Italian market. The results showed that about half of the samples analyzed presented benzo[a]pyrene (BaP) concentrations exceeding 2 μg/kg, which is proposed as a regulatory limit for dietary supplements.
Ali Anma, 2013 [47]	Bangladesh	Regulatory paper	This study investigated food safety and public health issues in Bangladesh. The study showed that use of formalin and DDT in foods is a crucial problem in Bangladesh. Supermarkets openly sell fruits, fishes, and vegetables that have been treated with formalin to keep them fresh. In Bangladesh, DDT is commonly used in dried fish (locally called as *sutki*) processing.
Hossain MM, et al., 2008 [48]	Bangladesh	Research article	In this study, the following chemicals were found to be used in foods and foodstuffs: calcium carbide, sodium cyclamate, cyanide, urea (a nitrogen-release fertilizer), and formalin. The sellers/producers mentioned the following reasons: for their use of harmful chemicals: to make the product more lucrative (40%), to extend the product’s shelf life (32%), to substitute for unavailable natural raw materials (natural raw materials were not always available) (16%), consumer demand (8%), or because the adulterated raw materials were cheaper than natural goods (4%).

### Food adulteration

In 9 (11%) of full-text articles included in this review, food adulteration has been discussed as a major public health risk associated with food safety issues in the food market. Most of the foodstuffs in the market are adulterated in varying degrees. Chemicals (like urea fertilizer, artificial color flavors, textile dye, formalin, chlorofluorocarbon; DDT powder, sodium bicarbonate, neutralizers, detergents, hydrogen peroxide, caustic soda, sodium chloride, boric acid, ammonium sulfate, sorbitol, metanil yellow, ultramarine blue, rhodamine B., maleic anhydride, copper chlorophyll, dimethyl/diethyl yellow, argemone oil, burnt mobil, and burnt oil); items which are not the genuine component of foods (like potato smash, cow’s fat and intestine in ghee, water in milk, sugar in honey, etc.); poor-quality products; and physical or inert agents (like saw dust and brick powder) are the commonest adulterants added to different food items (Table 5).

**Table 5:** Summary of full-text articles which reported food adulteration as a public risk in food marketing

Authors	Country/region	Article type	Main message/findings
Ali Anma, 2013 [47]	Bangladesh	Regulatory paper	This study investigated food safety and public health issues in Bangladesh. The study pointed out that most of the foodstuffs, be it manufactured or processed, are adulterated in varying degrees. The puffed rice is contaminated by using the urea fertilizer to make it whiter and bigger in size. Ghee is adulterated rotten milk, palm oil, soybean, animal or vegetable fat, potato paste, and with artificial color flavors.
Nasreen S and Ahmed T, 2014 [49]	Bangladesh	Research article	This study investigated the magnitude of food adulteration during 1995–2011 and consumer awareness in Dhaka City. The study reported that 40–54% of daily-consumed food was adulterated during 1995–2011. More than 35 food items were commonly adulterated. Some of the hazardous adulterants were white eggs of farm hens colored red with textile dye to sell as local hen eggs; inject formalin through the gills; or dip fishes in water treated with chemicals, such as chlorofluorocarbon; DDT powder to prevent rotting; textile dye in sweetened curd; toxic chemical, potato smash, cow’s fat and intestine in ghee; chemicals, color, burnt mobil from rail locomotives, and burnt oil from electric transformer in edible oils; urea in rice to make it whiter; and many more others.
Chanda T, et al., 2012 [50]	Bangladesh	Research article	This study aimed to detect the type of adulterants and preservatives added to the incoming fluid milk from rural areas to the Barisal, Bangladesh. The results indicated that 100% of the milk samples were adulterated with water. Cane sugar, powdered milk, and starch were detected as 26.0, 14.0, and 12.0% in the milk samples, respectively. Out of all samples, 10.0% was adulterated with formalin and 20.0% with sodium bicarbonate.
Singuluri H and Sukumaran M, 2014 [51]	India	Research article	The study assessed adulteration of natural milk with various illegal substances. The results pointed out that sucrose and skim milk powder were present in 22% and 80% of the milk samples respectively. Urea, neutralizers, and salt were present in 60%, 26%, and 82% of the milk samples respectively. Formalin, detergents, and hydrogen peroxide were present in 32%, 44%, and 32% of the milk samples obtained.
Barham GS, et al., 2014 [52]	Pakistan	Research article	This study examined various adulterants of milk in Pakistan. The study found that water (73%), detergent (32%), cane sugar (22%), caustic soda (20%), rice flour (17%), sodium chloride and skimmed milk powder (15%), hydrogen peroxide (13%), starch (12%), formalin (11%), urea and vegetable oil (10%), boric acid (8%), ammonium sulfate (6%), glucose (5%), sorbitol (4%), and arrowroot (1%) were found in milk samples.
Waghray K, et al., 2011 [53]	India	Research article	This study identified the adulteration in different food products available in the twin cities of Hyderabad and Secunderabad. The findings showed that chili powder samples showed the presence of metanil yellow (8%) added color (92%) and saw dust (48%). Dry ginger samples (8.33%) showed the presence of an unpermitted colored dye ultramarine blue. The sweet meat samples showed the presence of aluminum foil (4.3%) instead of silver foil. Coconut burfi samples contained unpermitted color orange II and cotton candy and floss candy showed the presence of rhodamine B. The total percentage of adulteration in the food samples was found to be 49.41%.
Peng G-J, et al., 2017 [54]	Taiwan	Research article	This study outlines the major cases of food adulteration that occurred in Taiwan between 2011 and 2015, including the adulteration of food additives with plasticizers, starch products with maleic anhydride, olive oil with copper chlorophyll, lard with recycled cooking oil, and processed soymilk curd with dimethyl/diethyl yellow.
Woldemariam HW and Abera BD, 2014 [55]	Ethiopia	Research article	This study investigated the extent of adulteration of selected foods in Bahir Dar, Ethiopia. The result showed that 6.7% of butter samples were adulterated with vegetable sources, mainly mashed potatoes; 8% of coffee powder samples were adulterated with roasted cereals; 15% of honey samples were adulterated with sugar or invert sugar; 1.3% of the red pepper powder samples were adulterated with brick powder; and 2.7% of edible oil samples contain argemone oil.
Assefa A, et al., 2013 [56]	Ethiopia	Research article	This study investigated the causes of dropsy in Addis Ababa, Ethiopia. The result indicated that 47 of the 280 edible oils analyzed were adulterated with argemone oil.

### Misuse of food additives

In this systematic review of literature, 11 of 81 (14%) full-text articles showed that misuse of food additives in the food market endangers public health. As reported in the included full-text articles, even though some food colorants and sweeteners are permitted to use such as sunset yellow FCF (SSYFCF), tartrazine, erythrosine, new coccine, ponceau, and saccharin (some may not be permitted based on countries food regulation), their concentration exceeded the prescribed limit. Moreover, use of non-permitted colorants and sweeteners such as rhodamine B, metanil yellow, orange II, malachite green, auramine, quinoline yellow, amaranth, carmoisine, Sudan dyes, and cyclamate (some may be permitted based on countries food regulation) is also commonly reported in the included studies (Table 6).

**Table 6:** Summary of full-text articles which reported misuse of food additives as a public risk in food marketing

Authors	Country/region	Article type	Main message/findings
Dixit S, et al., 2011 [57]	India	Research article	This study assessed usage pattern of synthetic food colors in different states of India. The results revealed that the majority of candyfloss, sugar toys, beverages, mouth fresheners, ice candy, and bakery product samples exceeded the prescribed limit. Non-permitted colors were mostly prevalent in candyfloss and sugar toy samples. Though sunset yellow FCF (SSYFCF) and tartrazine were the two most popular colors, many samples used a blend of two or more colors. The blend of SSYFCF and tartrazine exceeded the prescribed limit by a factor of 37 in one sample.
Tripathi M, et al., 2007 [58]	India	Research article	This study assessed use of synthetic colors in India. The study reported that 31% samples contained non-permitted colors. In urban areas, samples of crushed ice which are preferentially consumed by children population, the presence of Sunset Yellow FCF and Tartrazine was found to exceed the permissible limit by 8 and 20 times while in rural areas, Sunset Yellow FCF, Tartrazine, and Carmoisine exceeded the permissible limit by 23, 16, and 15 times, respectively. Non-permitted colors such as rhodamine B, metanil yellow, orange II, malachite green, auramine, quinoline yellow, amaranth, and Sudan dyes were identified in various foodstuffs.
Stevens LJ, et al., 2014 [59]	USA	Research article	This study assessed amounts of artificial food colors in commonly consumed beverages in the USA. The findings showed that most sweetened and artificially sweetened carbonated beverages, fruit drinks and punches, sports drinks, and energy drinks are dyed with either caramel color or artificial colors in widely varying amounts. Beverages (liquid and powdered) contained a wide range of concentrations of artificial food colors from 1.2 to 48 mg/240 ml.
Rao P, et al., 2004 [60]	India	Research article	This study assessed exposure to synthetic food colors of a selected population in Hyderabad, India. The study reported that children had an intake of solid food consumption in the range 2–465 g day^–1^ and liquid food consumption in the range 25–840 ml day^–1^ with added colors. Among the eight permitted colors in India, six were consumed by the subjects of the study. The intakes of some subjects exceeded the acceptable daily intake for colors such as tartrazine, sunset yellow, and erythrosine, which is 7.5, 2.5, and 0.1 mg kg^–1^ body weight, respectively.
Ashfaq N and Masud T, 2002 [61]	Pakistan	Research article	This study assessed artificial colors in different ready-to-eat foods in Rawalpindi, Pakistan. The results showed that quantities of the permitted coloring matter among the tested samples were found within the range of 18–220 ppm and 47.56% of the samples contained non-permitted food colors.
Jonnalagadda PR, et al., 2004 [62]	India	Research article	This study assessed type, extent, and use of colors in ready-to-eat (RTE) in Hyderabad, India. The results showed that 90% of the samples contained permitted colors, 2% contained a combination of permitted and non-permitted colors, and 8% contained only non-permitted colors. However, in RTE foods with permitted colors, 73% exceeded 100 ppm. Among the permitted colors, tartrazine was the most widely used color followed by sunset yellow. The maximum concentration of colors was detected in sweet meats (18 767 ppm), non-alcoholic beverages (9450 ppm), miscellaneous foods (6106 ppm), and hard-boiled sugar confectioneries (3811 ppm). Among the non-permitted colors found, rhodamine was most commonly used.
Tsai C-F, et al., 2015 [63]	Taiwan	Research article	This study determined synthetic dyes in chili powders and syrup-preserved fruits purchased from retail establishments in Taipei City, Taiwan. The results showed that three legal food dyes, tartrazine, and/or sunset yellow FCF, and/or new coccine, are present in some syrup-preserved fruits. Amaranth, an illegal food dye is found in an imported syrup-preserved fruit.
Moradi-Khatoonabadi Z, et al., 2015 [64]	Iran	Research article	This study assessed synthetic food colors in foods from restaurants in Tehran, Iran. Of the total 573 samples, 52% were positive for at least one color. The most prevalent colors were tartrazine, quinoline yellow, and sunset yellow, with 44%, 9.1%, and 8.4% of the samples testing positive for these colors, respectively. Carmoisine and ponceau were both detected only in 0.5% of the positive samples and found only in saffron solution.
Saleem N and Umar ZN, 2013 [65]	Pakistan	Research article	This study assessed the type of food colors added to various food products especially those vended at or near different educational institutes of Karachi City, Pakistan. The results revealed that some foods manufactured locally contained non-permitted colors. About 11% branded and 44% unbranded food items, respectively, were found with not permitted colors for human consumption. Similarly, 4% branded and 30% unbranded beverages were found unfit due to the presence of prohibited colors.
Petigara Harp B, et al., 2013 [66]	USA	Research article	This survey assessed color additives in food products purchased from retail stores in Washington, DC, and surrounding Maryland counties. A survey of 44 food products, including beverages, frozen treats, powder mixes, gelatin products, candies, icings, jellies, spices, dressings, sauces, baked goods, and dairy products, found total color additives ranging from 1.9 to 1221 mg/kg.
Sood M, 2014 [67]	Indonesia	Field inspection	This field inspection on imported processed food products in Indonesia found processing food products that are not in accordance with the provisions. Some processing food products contain harmful substances such as formaldehyde, rhodamine B, saccharin, benzoic acid, methanol, yellow, and cyclamate, and preservatives and other harmful dyes.

### Mislabeling

Mislabeling of food products has been mentioned as a major public health risk associated with food safety in the food market in 17 of 81 (21%) full-text articles included in this review. All of the 17 studies reported that significant proportion of food samples collected from supermarkets, food stores, shops, and restaurants were genetically identified as entirely different species from that identified on the product labels, and therefore were considered as mislabeled. The studies witnessed that seafood is the most commonly mislabeled food product (Table 7).

**Table 7:** Summary of full-text articles which reported mislabeling as a public risk in food marketing

Authors	Country/region	Article type	Main message/findings
Miller DD and Mariani S, 2010 [68]	Ireland	Research article	This study collected food samples from supermarkets, shops, and restaurants in Dublin, Ireland to assess labeling and transparency in the European seafood industry. The assessment showed that 39 out of 156 (25%) samples were genetically identified as entirely different species from that identified on the product labels, and therefore were considered as mislabeled. More significantly, 28 out of 34(82.4%) smoked fish samples were found to be mislabeled.
Jacquet JL and Pauly D, 2008 [69]	USA	Research article	This paper examines the extent and consequences of renaming and mislabeling seafood, with particular attention to the USA, where 80% of the seafood is imported and more than one-third of all fish are mislabeled.
Armani A, et al., 2012	Italy	Research article	This survey assessed label compliance of jellyfish products sold on the Italian market. The survey found many shortfalls including the presence of a trade name referring to vegetables or a lack of an unequivocal specification of ingredients.
Armani A, et al., 2013	Italy and China	Research article	Forensically informative nucleotide sequencing (FINS) of a short mitochondrial COI gene fragment revealed 100% of the sample of ready-to-eat jellyfish food products in Italy and China were mislabeled.
Chin TC, et al., 2016 [70]	Malaysia	Research article	This study detected mislabeled seafood products in Malaysia by DNA barcoding. A total of 62 seafood samples, either raw, frozen, or variously processed, were collected from commercial sources in Malaysia. The DNA targets were successfully amplified and sequenced from 81% of seafood samples. Among these samples, 16% were found to have been mislabeled at source.
Nagalakshmi K, et al., 2016 [71]	India	Research article	This study found out the level of seafood mislabeling prevailing in India using DNA barcoding. A total of 100 seafood samples including fresh, frozen, ready-to-cook, ready-to-eat, and canned products were collected. The results revealed 22% of seafood mislabeling prevailing in Indian domestic market.
Galal-Khallaf A, et al., 2014 [72]	Egypt	Research article	This study assessed the labeling status of Egyptian fish fillets. DNA barcoding was applied to ascertaining species in fish fillets (tilapia, Nile perch, and panga) purchased from Egyptian markets. Ninety commercial samples were analyzed. Sequencing of a short fragment of mitochondrial cytochrome oxidase I (COI) gene revealed 33.3% species substitution in the fish fillets analyzed, 50% Nile perch (*Lates niloticus*) and 50% basa fish (*Pangasius bocourti*) being replaced by imported Vietnamese tra fish (*Pangasianodon hypophthalmus*).
Cawthorn D-M, et al., 2012 [73]	South Africa	Research article	This study investigated incidence of fish species misrepresentation and substitution on the South African market. The results showed that 10 of 108 (9%) samples from wholesalers and 43 of 140 (31%) from retailers were identified as different species to the ones indicated at the point of sale.
Di Pinto A, et al., 2015 [74]	Italy	Research article	This study investigated processed-meat products from Italian markets in order to verify any species substitution or mislabeling. The results revealed a high substitution rate among the meat products, highlighting a mislabeling rate of 57%, and consequently, considerable discordance with the indications on the labels.
Carvalho DC, et al., 2017 [75]	Brazil	Research article	This study analyzed twenty-two processed cod products purchased from supermarkets, local stores, fast food outlets, and one restaurant in the city of Belo Horizonte, Brazil. A mixture of two or more species was found within 31% of all products and 41% mislabeling was reported within highly processed cod products.
Garcia-Vazquez E, et al., 2010 [76]	Spain and Greek	Research article	DNA analysis of hake products commercialized in Spanish and Greek market chains has demonstrated more than 30% mislabeling, on the basis of species substitution. Tails and fillets were more mislabeled than other products, such as slices and whole pieces. African species were substitute species for products labeled as American and European species.
Staffen CF, et al., 2017 [77]	Brazil	Research article	This study assessed labeling of fish products in a popular tourist destination in Brazil. A DNA barcoding of 65 samples from fisheries and 80 from restaurants revealed that 30% of mislabeled samples in fisheries and 26% in restaurants.
Muñoz-Colmenero M, et al., 2017 [78]	Spain, USA, and Canada	Research article	This study assessed mislabeling in salmon products from two regions, Northwest of America and Northwest of Spain. A DNA barcoding of samples indicated that the Spanish and Northwest American samples were mislabeled 6% and 23.8% respectively.
Muñoz-Colmenero M, 2016 [79]	Spain	Research article	This study authenticated the species of fish marketed in Spain. DNA sequences of 245 fish samples revealed greater than 7% mislabeling.
Bosko SA, et al., 2018 [80]	USA	Research article	This study tested 80 catfish samples collected from restaurants, grocery stores, and fish markets in the USA tested with real-time PCR. A DNA barcoding of samples showed that 7 of the 80 catfish products were found to be substituted with Pangasiidae species for a mislabeling rate of 9%. This included 5 of the 40 restaurant samples and 2 of the 32 grocery store samples.
Christiansen H, et al., 2018 [81]	Belgium	Research article	This study assessed seafood substitution and mislabeling in Brussels’ restaurants and canteens. A DNA barcoding revealed that 31.1% of the samples were mislabeled, with mislabeling present in all types of vendors. Cod and sole were the most frequently sampled and were also mislabeled regularly (13.1% and 11.1%). Bluefin tuna was substituted almost always (95% mislabeling).
Galal-Khallaf A, et al., 2002 [82]	Egypt and Spain	Research article	This study is a PCR-based assessment of shellfish traceability and sustainability in seafood markets. The results found that 17.2% and 15.2% products were mislabeled in Egypt and Spain, respectively.

### Genetically modified foods

In this systematic review of literature, 4 of 81 (5%) of the included full-text articles discussed that GM foods are becoming an increasing public health risk. Hypertension, stroke, diabetes, obesity, lipoprotein metabolism disorder, Alzheimer’s, Parkinson’s, multiple sclerosis, hepatitis C, end-stage renal disease, acute kidney failure, cancers of the thyroid/liver/bladder/pancreas/kidney, myeloid leukemia, diarrhea, vomiting, difficulty in breathing, respiratory problems, hormonal imbalances and susceptibility to infection or immunosuppression, allergenic or rashes, and chemical toxicity are health problems reported in the included full-text articles (Table 8).

**Table 8:** Summary of full-text articles which reported genetically modified foods as a public risk in food marketing

Authors	Country/region	Article type	Main message/findings
Swanson NL, et al., 2014 [83]	USA	Research article	This study found that the Pearson correlation coefficients are highly significant (< 10^−4^) between the percentage of GE corn and soy planted in the USA and hypertension (*R* = 0.961), stroke (*R* = 0.983), diabetes prevalence (*R* = 0.983), diabetes incidence (*R* = 0.955), obesity (*R* = 0.962), lipoprotein metabolism disorder (*R* = 0.955), Alzheimer’s (*R* = 0.937), Parkinson’s (*R* = 0.952), multiple sclerosis (*R* = 0.876), hepatitis C (*R* = 0.946), end-stage renal disease (*R* = 0.958), acute kidney failure (*R* = 0.967), cancers of the thyroid (*R* = 0.938), liver (*R* = 0.911), bladder (*R* = 0.945), pancreas (*R* = 0.841), kidney (*R* = 0.940), and myeloid leukemia (*R* = 0.889).
Pattron DD, 2005 [84]	Eastern Caribbean	Research article	This study investigated health implications associated with GM foods in Trinidad. The survey found that diarrhea, vomiting, rashes, difficulty in breathing, respiratory problems, hormonal imbalances, and susceptibility to infection or immunosuppression are common reported health problems associated with consuming GM foods. These medical claims were supported by medical certificates, diagnosis, treatment regimens, and physician letters and/or prescriptions. Foods consumed were validated against the list of known genetically modified foods
Bakshi A, 2003 [85]	General setting	Research article	There are concerns about the safety of genetically modified crops. The concerns are that they may contain allergenic substances due to introduction of new genes into crops. Another concern is that genetic engineering often involves the use of antibiotic-resistance genes as “selectable markers” and this could lead to production of antibiotic-resistant bacterial strains that are resistant to available antibiotics. This would create a serious public health problem. The genetically modified crops might contain other toxic substances (such as enhanced amounts of heavy metals).
Aris A and Leblanc S, 2011 [86]	Canada	Research article	This study highlighted the presence of pesticide-associated genetically modified foods in maternal, fetal, and non-pregnant women’s blood in Quebec, Canada. 3-MPPA and Cry1Ab toxins are clearly detectable and appear to cross the placenta to the fetus.

### Foods past their use-by dates

Six (7%) of the included full-text articles revealed that outdated or foods past their use-by dates are being sold in food stores, shops, and restaurants which are contributing huge public health and environmental problems (Table 9).

**Table 9:** Summary of full-text articles which reported foods past their use-by dates as a public risk in food marketing

Authors	Country/region	Article type	Main message/findings
Anyanwu RC and Jukes DJ, 1991 [87]	Nigeria	Research article	This study assessed food systems and food control in Nigeria. The results showed that foods are very poorly handled in the rural food system, with expired food being sold.
Burnett K, et al., 2015 [88]	Canada	Research article	This is an online survey gathered community input about retail and food purchasing experiences in northern Canada. Preliminary findings show that expired foods are of the top three concerns of food safety. Eighty-two percent stated that their store often or sometimes sold expired food.
Freedman DA and Bell BA, 2009 [89]	USA	Research article	This study investigated access to foods among an urban food insecure population in Nashville, USA. In this study, 10 of 37 (27%) of the participants reported that food stores in their neighborhood sell outdated foods.
Sood M, 2014 [67]	Indonesia	Field inspection	This field inspection on imported processed food products in Indonesia found processing food products that are not in accordance with the provisions. Expired food products are found sold in various markets, such as supermarkets, shops, and traditional markets, such products also circulated illegally entered and especially to the areas that have access to transportation that are difficult to reach.
Islam M, 2017 [36]	Bangladesh	Thesis	This study assessed bacteriological quality of street-vended and expired food items collected from different areas in Dhaka City, Bangladesh. Out of total 35 food samples (expired and street), enteric bacteria were found in 17 (48.6%) food samples containing *E. coli*, *Vibrio*, *Shigella*, and *Salmonella* species.
Kunyanga C, et al., 2011 [90]	Kenya	Research article	This study assessed characteristics of foods sold and consumed by vulnerable groups in Kenya. The study reported that it was possible for consumers to continue using the foods even after they had expired and were no longer able to meet the nutrition and health requirements at the levels declared on the labels.

## Discussion

This review identified that microbial contamination, chemical contamination, adulteration, misuse of food additives, mislabeling, genetically modified foods, and outdated foods are common public health risks related with food safety issues in the food market. In the food market, food can become contaminated in one country and cause health problems in another. These food safety issues cause exposure of consumers to biological, chemical, and physical hazards [91–95] so that endanger health of the public. The origin of food hazards can be described as a chain which commences on the source and continues with transportation, further processing steps, merchandising events and finally ends with the consumer [96–100]. Overall, this review suggested that food safety–related public health risks are more common in developing countries than developed countries. This can be justified that foods get easily contaminated with microbes due to the poor hygiene and sanitation in developing countries [101–104]. Moreover, hence the regulatory services are weak in developing countries, most food sellers may not comply with food hygiene and safety requirements or standards [105–107]. In developing countries, the legislation enforcement is still weak about administrating the concentration of harmful contaminants in the food [108, 109]. In addition, there is inadequate information and technology to detect fake and fraud products **[**110–112**]**.

This review identified that microbial contamination of foods in the food market is commonly reported in many studies. Different bacterial species and funguses were the commonest diseases causing pathogens identified [17–35, 113]. Failure to apply food safety strategies in every stage of the food supply chain, for example bad food handling practices, poor production process, poor agricultural practices, poor transportation system, poor marketing practices, and poor sanitation lead to microbial contamination of foods [114–118]. Moreover, fraud of foods such as adulteration, mislabeling, and selling of spoiled or expired foods are also causing microbial contamination [36, 119–122]. Microbial contamination of foods causes millions of diseases and thousands of deaths [123]. This review also shows that total coliforms, fecal coliforms, and different fungus were commonly reported in developing countries than developed countries. This might be due to the fact that fecal contamination of foods and the environment is common in developing countries due to poor sanitation condition [124–126]. Moreover, the temperature and air system of food storage areas are not well regulated in developing countries. This situation creates favorable condition for molds. On the other hand, different Campylobacter species were reported in developed countries. This might be due to the fact that advancement of molecular techniques to identify these microorganisms. Developing countries lack specialized cultivation techniques to culture these organisms [127]. The standard culture–based technique, which is a predominant detection method in developing countries, is not effective for Campylobacter species [128–130].

Contamination of foods with hazardous chemicals has been reported as a major public health concern associated with the food market in individual studies included in this review [37–46, 48, 131–133]. The phases of food processing, packaging, transportation, and storage are significant contributors to food contamination [109]. Food contaminants include environmental contaminants, food processing contaminants, unapproved adulterants and food additives, and migrants from packaging materials. Environmental contaminants are impurities that are either introduced by human or occurring naturally in water, air, or soil. Food processing contaminants include those undesirable compounds, which are formed in the food during baking, roasting, canning, heating, fermentation, or hydrolysis. The direct food contact with packaging materials can lead to chemical contamination due to the migration of some harmful substances into foods. Use of unapproved or erroneous additives may result in food contamination [134–138]. Chemical contamination of foods is responsible millions of cases of poisoning with thousands of hospitalizations and deaths each year [139].

Nine of the full-text articles included in this review reported that food adulteration is a major public health risk associated with food safety issues in the food market. Chemicals, items which are not the genuine component of foods, poor-quality products, and physical or inert agents are the commonest adulterants added [47, 49–56]. Food adulteration involves intentional or unintentional addition of useless, harmful, unnecessary chemical, physical, and biological agents to food which decreases the quality of food. It also includes removal of genuine components and processing foods in unhygienic way **[**119, 140]. However, removal of genuine components of food is not considered in this review. Food is adulterated to increase the quantity and make more profit, which is economically motivated adulteration [141–143]. Chemicals which are being used as adulterants have a wide range of serious effects on the health of consumers including cancer [119, 144–147].

In this systematic review of literature, 11 of the full-text articles reported that misuse of food additives in the food market endangers public health **[**57–67]. Food additive is any substance not normally consumed as a food by itself; not normally used as a typical ingredient of the food (whether or not it has nutritive value); and added intentionally to food for a technological purpose in the production process for the purpose of maintaining a food’s nutritional quality, for example by preventing the degradation of vitamins, essential amino acids, and unsaturated fats; extending the shelf life of a product, for example by preventing microbial growth; and maintaining and improving a product’s sensory properties, such as texture, consistency, taste, flavor, and color; Being able to provide products [148, 149]. Substances generally recognized as safe (GRAS) can be used as food additives [150, 151]; however, misuse of substances such as using more than the maximum allowable concentration; using non-permitted substances; and blending of permitted and non-permitted substances together causes health hazards [152, 153].

Mislabeling of food products has been mentioned as a major public health risk associated with food safety in the food market in 17 of the full-text articles included in this review **[**68–82, 154]. Mislabeling of food products includes false advertising, deliberately or accidentally leaving out ingredients, not listing potential health effects, and claiming a food contains ingredients that it does not for financial gain with the intent of deceiving the consumer regarding what is actually in the package [155]. These acts of fraud have increased overtime as different parties such as manufacturers, co-packers, distributors, and others along the chain of distribution involve in the national or international trade. Mislabeling leads to cross-contamination, poor food quality, degradation of nutrients, and even adverse effects on human health, serious financial, and legal consequences [69, 154].

In this systematic review, we identified that GM foods are becoming an increasing public health risk. The included full-text articles reported that a wide range of health consequences associated with consumption of GM foods [83–86]. Possible hazards of GM foods include the potential for pleiotropic and insertional effects (silencing of genes, changes in their level of expression or, potentially, the turning on of existing genes that were not previously being expressed), effects on animal and human health resulting from the increase of anti-nutrients, potential effects on human health resulting from the use of viral DNA in plants, possible transfer of antibiotic-resistant genes to bacteria in gastrointestinal tract, and possible effects of GM foods on allergic responses [156–161]. However, the health effects of genetically modified foods are still debatable. Different lab-animal-based studies reported that there is no safety difference between GM and non-GM foods or the health concerns are not confirmed well [162–165]. Some others argue that despite the advances in food crop agriculture, the current world situation is still characterized by massive hunger and chronic malnutrition, representing a major public health problem. Biofortified GM crops have been considered an important and complementary strategy for delivering naturally fortified staple foods to malnourished populations [164].

This review revealed that foods past their use-by dates in the food market are major threats for consumers. This malpractice is more common in less developed countries and rural markets **[**36, 67, 87–90]. Growth of microorganisms in expired foods is very common. Most of these microorganisms are pathogenic and some microorganisms produce toxic substances as they develop [36, 121, 166–169].

## Limitation of the review

We entirely relied on electronic databases to search relevant articles. We did not include articles available in hard copy. We believed we could get more relevant articles if we had access to hard prints.

## Conclusion

This systematic literature review identified common food safety–related public health risks in the food market. The results imply that the local and international food marketing continues to have significant impacts on health of the public. The food market increases internationalization of health risks as the food supply chains cross multiple national borders. Therefore, effective national food control systems are essential to protect the health and safety of the public. Countries have to implement and enforce risk-based food control strategies. Countries need also assure the safety and quality of their foods entering international trade and ensure that imported foods conform to national requirements. Moreover, food producers and retail sectors have to respect the national food safety guideline and have to work to protect the safety of their customers Additional file 1.

## List of full text articles included in the review

The full text articles included in this review are attached as a supplementary file (see supplementary file).

## Supplementary information

**Additional file 1.** List of full text articles included in the review.

## Data Availability

All the extracted data are included in the manuscript.

## Abbreviations

DALYs: Disability adjusted life years
GM: Genetically modified foods
GRAS: Substances generally recognized as safe
JBI: Joanna Briggs Institute
MMAT: Mixed methods appraisal tool
PICo: Population, phenomena of interest, and context
