# Supplementary material for: Public health risks related to food safety issues in the food market: a systematic literature review
Source: Environ Health Prev Med. 2019 Nov 30;24:68. doi: 10.1186/s12199-019-0825-5 (PMC6885314; doi:10.1186/s12199-019-0825-5)
Supplement: Supplementary file 1 — Additional file 1. List of full text articles included in the review. [file 12199_2019_825_MOESM1_ESM.docx]

**List of full text articles included in the review**

1. **Articles on microbial contamination of foods**

**Gabriel AA, et al, 2007 (Research article)**

Gabriel AA, Berja MC, Estrada AMP, Lopez MGAA, Nery JGB, Villaflor EJB. Microbiology of retail mung bean sprouts vended in public markets of National Capital Region, Philippines. Food Control. 2007;18(10):1307-13.

**Adeyanju GT & Ishola O, 2014 (Research article)**

Adeyanju GT, Ishola O. Salmonella and Escherichia coli contamination of poultry meat from a processing plant and retail markets in Ibadan, Oyo State, Nigeria. Springerplus. 2014;3(1):139.

**Giammanco GM, et al, 2011 (Research article)**

Giammanco GM, Pepe A, Aleo A, D'Agostino V, Milone S, Mammina C. Microbiological quality of Pecorino Siciliano" primosale" cheese on retail sale in the street markets of Palermo, Italy. New Microbiologica. 2011;34(2):179-85.

**Zhao C, et al, 2001 (Research article)**

Zhao C, Ge B, De Villena J, Sudler R, Yeh E, Zhao S, et al. Prevalence of Campylobacter spp., Escherichia coli, and Salmonella serovars in retail chicken, turkey, pork, and beef from the Greater Washington, DC, area. Appl Environ Microbiol. 2001;67(12):5431-6.

**Cárdenas C, et al, 2013 (Research article)**

Cárdenas C, Molina K, Heredia N, García S. Evaluation of microbial contamination of tomatoes and peppers at retail markets in Monterrey, Mexico. Journal of food protection. 2013;76(8):1475-9.

**Filiousis G, et al, 2009 (Short Communication)**

Filiousis G, Johansson A, Frey J, Perreten V. Prevalence, genetic diversity and antimicrobial susceptibility of Listeria monocytogenes isolated from open-air food markets in Greece. Food control. 2009;20(3):314-7.

**Pérez-Rodríguez F, et al, 2010 (Research article)**

Pérez-Rodríguez F, Castro R, Posada-Izquierdo G, Valero A, Carrasco E, García-Gimeno R, et al. Evaluation of hygiene practices and microbiological quality of cooked meat products during slicing and handling at retail. Meat science. 2010;86(2):479-85.

**Yagoub SO, 2009 (Research article)**

Yagoub SO. Isolation of Enterobacteriaceae and Pseudomonas spp. from raw fish sold in fish market in Khartoum state. African Journal of Bacteriology Research. 2009;1(7):085-8.

**Kumari S & Sarkar PK, 2014 (Research article)**

Kumari S, Sarkar PK. Prevalence and characterization of Bacillus cereus group from various marketed dairy products in India. Dairy science & technology. 2014;94(5):483-97.

**Domınguez C, et al, 2002 (Research article)**

Domınguez C, Gomez I, Zumalacarregui J. Prevalence of Salmonella and Campylobacter in retail chicken meat in Spain. International Journal of Food Microbiology. 2002;72(1-2):165-8.

**Vantarakis A, et al, 2011 (Research article)**

Vantarakis A, Affifi M, Kokkinos P, Tsibouxi M, Papapetropoulou M. Occurrence of microorganisms of public health and spoilage significance in fruit juices sold in retail markets in Greece. Anaerobe. 2011;17(6):288-91.

**Heredia N, et al, 2001 (Research note)**

Heredia N, Garcia S, Rojas G, Salazar L. Microbiological condition of ground meat retailed in Monterrey, Mexico. Journal of food protection. 2001;64(8):1249-51.

**Nel S, et al, 2004 (Research article)**

Nel S, Lues J, Buys E, Venter P. Bacterial populations associated with meat from the deboning room of a high throughput red meat abattoir. Meat science. 2004;66(3):667-74.

**Elson R, et al, 2004 (Research article)**

Elson R, Burgess F, Little C, Mitchell R, Services LACooR, Agency tHP. Microbiological examination of ready‐to‐eat cold sliced meats and pâté from catering and retail premises in the UK. Journal of applied microbiology. 2004;96(3):499-509.

**Hosseini A. 2011 (Research article)**

Hosseini A. The prevalence of bacterial contamination of table eggs from retails markets by Salmonella spp., Listeria monocytogenes, Campylobacter jejuni and Escherichia coli in Shahrekord, Iran. Jundishapur Journal of Microbiology. 2011;4(4):249.

**Banerjee M and Sarkar PK, 2003 (Research article)**

Banerjee M, Sarkar PK. Microbiological quality of some retail spices in India. Food Research International. 2003;36(5):469-74.

**Vindigni SM, et al, 2007 (Research article)**

Vindigni SM, Srijan A, Wongstitwilairoong B, Marcus R, Meek J, Riley PL, et al. Prevalence of foodborne microorganisms in retail foods in Thailand. Foodborne Pathogens and Disease. 2007;4(2):208-15.

**Simforian E, et al, 2015 (Research article)**

Simforian E, Nonga H, Ndabikunze B. Assessment of microbiological quality of raw fruit juice vended in Dar es Salaam City, Tanzania. Food Control. 2015;57:302-7.

**Mailafia S, et al, 2017 (Research article)**

Mailafia S, God’spower Richard Okoh HO, Olabode K, Osanupin R. Isolation and identification of fungi associated with spoilt fruits vended in Gwagwalada market, Abuja, Nigeria. Veterinary world. 2017;10(4):393.

**Hunter PR, et al, 1994 (Research article)**

Hunter PR, Hornby H, Campbell CK, Browne KF. Isolation of food spoilage yeasts from salads purchased from delicatessens. British Food Journal. 1994;96(3):23-5.

**Islam M, 2017 (Thesis)**

Islam M. Study on Bacteriological Quality of Street-Vended and Expired Food Items Collected from Different Areas in Dhaka City, Bangladesh: East West University; 2017.

1. **Articles on chemical contamination of foods**

**Bai Y, et al, 2006 (Research article)**

Bai Y, Zhou L, Wang J. Organophosphorus pesticide residues in market foods in Shaanxi area, China. Food Chemistry. 2006;98(2):240-2.

**Othman ZAA, 2010 (Research article)**

Othman ZAA. Lead contamination in selected foods from Riyadh city market and estimation of the daily intake. Molecules. 2010;15(10):7482-97.

**Zaied C, et al, 2013 (Research article)**

Zaied C, Abid S, Hlel W, Bacha H. Occurrence of patulin in apple-based-foods largely consumed in Tunisia. Food control. 2013;31(2):263-7.

**Schecter A, et al, 2010 (Research article)**

Schecter A, Colacino J, Haffner D, Patel K, Opel M, Päpke O, et al. Perfluorinated compounds, polychlorinated biphenyls, and organochlorine pesticide contamination in composite food samples from Dallas, Texas, USA. Environmental health perspectives. 2010;118(6):796-802.

**Onianwa P, et al, 2001 (Research article)**

Schecter A, Colacino J, Haffner D, Patel K, Opel M, Päpke O, et al. Perfluorinated compounds, polychlorinated biphenyls, and organochlorine pesticide contamination in composite food samples from Dallas, Texas, USA. Environmental health perspectives. 2010;118(6):796-802.

**Vinci RM, et al, 2015 (Research article)**

Vinci RM, Jacxsens L, De Meulenaer B, Deconink E, Matsiko E, Lachat C, et al. Occurrence of volatile organic compounds in foods from the Belgian market and dietary exposure assessment. Food control. 2015;52:1-8.

**Tittlemier SA, et al, 2004 (Research article)**

Tittlemier SA, Forsyth D, Breakell K, Verigin V, Ryan JJ, Hayward S. Polybrominated diphenyl ethers in retail fish and shellfish samples purchased from Canadian markets. Journal of agricultural and food chemistry. 2004;52(25):7740-5.

**Onianwa P, et al, 2000 (Short communication)**

Onianwa P, Lawal J, Ogunkeye A, Orejimi B. Cadmium and nickel composition of Nigerian foods. Journal of Food Composition and Analysis. 2000;13(6):961-9.

**Radwan MA and Salama AK, 2006 (Research article)**

Radwan MA, Salama AK. Market basket survey for some heavy metals in Egyptian fruits and vegetables. Food and Chemical Toxicology. 2006;44(8):1273-8.

**Ali MH and Al-Qahtani KM, 2012 (Research article)**

Ali MH, Al-Qahtani KM. Assessment of some heavy metals in vegetables, cereals and fruits in Saudi Arabian markets. The Egyptian Journal of Aquatic Research. 2012;38(1):31-7.

**NIE Ji-yun, et al, 2016 (Research article)**

NIE Ji-Yun, KUANG L-x, LI Z-x, XU W-h, Cheng W, CHEN Q-s, et al. Assessing the concentration and potential health risk of heavy metals in China's main deciduous fruits. Journal of integrative agriculture. 2016;15(7):1645-55.

**Vinci RM, et al, 2012 (Research article)**

Vinci RM, Jacxsens L, Van Loco J, Matsiko E, Lachat C, de Schaetzen T, et al. Assessment of human exposure to benzene through foods from the Belgian market. Chemosphere. 2012;88(8):1001-7.

**Moret S, et al, 2010 (Research article)**

Moret S, Purcaro G, Conte LS. Polycyclic aromatic hydrocarbons (PAHs) levels in propolis and propolis- based dietary supplements from the Italian market. Food Chemistry. 2010;122(1):333-8.

1. **Articles on adulteration of foods**

**Ali Anma, 2013 (Regulatory paper)**

Ali ANMA. Food safety and public health issues in Bangladesh: a regulatory concern. European Food and Feed Law Review. 2013:31-40.

**Nasreen S & Ahmed T, 2014 (Research article)**

Nasreen S, Ahmed T. Food adulteration and consumer awareness in Dhaka City, 1995-2011. Journal of health, population, and nutrition. 2014;32(3):452.

**Chanda T, et al, 2012 (Research article)**

Chanda T, Debnath G, Hossain M, Islam M, Begum M. Adulteration of raw milk in the rural areas of Barisal district of Bangladesh. Bangladesh Journal of Animal Science. 2012;41(2):112-5.

**Singuluri H & Sukumaran M, 2014 (Research article )**

Singuluri H, Sukumaran M. Milk adulteration in Hyderabad, India-a comparative study on the levels of different adulterants present in milk. Journal of Chromatography & Separation Techniques. 2014;5(1):1.

**Barham GS, et al, 2014 (Research article)**

Barham GS, Khaskheli M, Soomro AH, Nizamani ZA. Extent of extraneous water and detection of various adulterants in market milk at Mirpurkhas, Pakistan. J Agri Vet Sci. 2014;7(3):83-9.

**Waghray K, et al, 2011 (Research article )**

Waghray K, Gulla S, Thyagarajan P, Vinod G. Adulteration pattern in different food products sold in the twin cities of Hyderabad and Secunderabad-India. Journal of Dairying Foods & Home Sciences. 2011;30(2).

**Peng G-J, et al, 2017 (Research article)**

Peng G-J, Chang M-H, Fang M, Liao C-D, Tsai C-F, Tseng S-H, et al. Incidents of major food adulteration in Taiwan between 2011 and 2015. Food Control. 2017;72:145-52.

**Woldemariam HW & Abera BD, 2014 (Research article)**

Woldemariam HW, Abera BD. The Extent of Adulteration of Selected Foods at Bahir Dar, Ethiopia. International Journal of Interdisciplinary Research. 2014;1(6):1-6.

**Assefa A, et al, 2013 (Research article)**

Assefa A, Teka F, Guta M, Melaku D, Naser E, Tesfaye B, et al. Laboratory investigation of epidemic dropsy in Addis Ababa, Ethiopia. Ethiopian medical journal. 2013:21-32.

1. **Articles on food additives**

**Dixit S, et al, 2011 (Research article)**

Dixit S, Purshottam S, Khanna S, Das M. Usage pattern of synthetic food colours in different states of India and exposure assessment through commodities preferentially consumed by children. Food Additives & Contaminants: Part A. 2011;28(8):996-1005.

**Tripathi M, et al, 2007 (Research article)**

Tripathi M, Khanna SK, Das M. Surveillance on use of synthetic colours in eatables vis a vis Prevention of Food Adulteration Act of India. Food Control. 2007;18(3):211-9.

**Stevens LJ, et al, 2014 (Research article)**

Stevens LJ, Burgess JR, Stochelski MA, Kuczek T. Amounts of artificial food colors in commonly consumed beverages and potential behavioral implications for consumption in children. Clinical pediatrics. 2014;53(2):133-40.

**Rao P, et al, 2004 (Research article)**

Rao P, Bhat R, Sudershan R, Krishna T, Naidu N. Exposure assessment to synthetic food colours of a selected population in Hyderabad, India. Food additives and contaminants. 2004;21(5):415-21.

**Ashfaq N & Masud T, 2002 (Research article)**

Ashfaq N, Masud T. Surveillance on artifical colours in different ready to eat foods. Pakistan J Nutr. 2002;5:223-5.

**Jonnalagadda PR, et al, 2004 (Research article)**

Jonnalagadda PR, Rao P, Bhat RV, Nadamuni Naidu A. Type, extent and use of colours in ready‐to‐eat (RTE) foods prepared in the non‐industrial sector–a case study from Hyderabad, India. International journal of food science & technology. 2004;39(2):125-31.

**Tsai C-F, et al, 2015 (Research article)**

Tsai C-F, Kuo C-H, Shih DY-C. Determination of 20 synthetic dyes in chili powders and syrup-preserved fruits by liquid chromatography/tandem mass spectrometry. Journal of food and drug analysis. 2015;23(3):453-62.

**Moradi-Khatoonabadi Z, et al, 2015 (Research article)**

Moradi-Khatoonabadi Z, Amirpour M, AkbariAzam M. Synthetic food colours in saffron solutions, saffron rice and saffron chicken from restaurants in Tehran, Iran. Food Additives & Contaminants: Part B. 2015;8(1):12-7.

**Saleem N & Umar ZN, 2013 (Research article)**

Saleem N, Umar ZN. Survey on the use of synthetic Food Colors in Food Samples procured from different educational institutes of Karachi city. Journal of tropical life science. 2013;3(1):1-7.

**Petigara Harp B, et al, 2013 (Research article)**

Petigara Harp B, Miranda-Bermudez E, Barrows JN. Determination of seven certified color additives in food products using liquid chromatography. Journal of agricultural and food chemistry. 2013;61(15):3726- 36.

**Sood M, 2014 (Field inspection)**

Sood M. The Supervision of Government on Implementation of Import of Processed Food Products in Effort of Legal Protection for Consumers. JL Pol'y & Globalization. 2014;25:72.

1. **Articles on mislabeling**

**Miller DD & Mariani S, 2010 (Research article)**

Miller DD, Mariani S. Smoke, mirrors, and mislabeled cod: poor transparency in the European seafood industry. Frontiers in Ecology and the Environment. 2010;8(10):517-21.

**Jacquet JL & Pauly D, 2008 (Research article)**

Jacquet JL, Pauly D. Trade secrets: renaming and mislabeling of seafood. Marine Policy. 2008;32(3):309- 18.

**Armani A, et al, 2012 (Research article)**

Armani A, D’Amico P, Castigliego L, Sheng G, Gianfaldoni D, Guidi A. Mislabeling of an “unlabelable” seafood sold on the European market: The jellyfish. Food Control. 2012;26(2):247-51.

**Armani A, et al, 2013 (Research article)**

Armani A, Tinacci L, Giusti A, Castigliego L, Gianfaldoni D, Guidi A. What is inside the jar? Forensically informative nucleotide sequencing (FINS) of a short mitochondrial COI gene fragment reveals a high percentage of mislabeling in jellyfish food products. Food Research International. 2013;54(2):1383-93.

**Chin TC, et al, 2016 (Research article)**

Chin TC, Adibah A, Hariz ZD, Azizah MS. Detection of mislabelled seafood products in Malaysia by DNA barcoding: Improving transparency in food market. Food Control. 2016;64:247-56.

**Nagalakshmi K, et al, 2016 (Research article)**

Nagalakshmi K, Annam P-K, Venkateshwarlu G, Pathakota G-B, Lakra WS. Mislabeling in Indian seafood: An investigation using DNA barcoding. Food control. 2016;59:196-200.

**Galal-Khallaf A, et al, 2014 (Research article)**

Galal-Khallaf A, Ardura A, Mohammed-Geba K, Borrell YJ, Garcia-Vazquez E. DNA barcoding reveals a high level of mislabeling in Egyptian fish fillets. Food Control. 2014;46:441-5.

**Cawthorn D-M, et al, 2012 (Research article)**

Cawthorn D-M, Steinman HA, Witthuhn RC. DNA barcoding reveals a high incidence of fish species misrepresentation and substitution on the South African market. Food Research International. 2012;46(1):30-40.

**Di Pinto A, et al, 2015 (Research article)**

Di Pinto A, Bottaro M, Bonerba E, Bozzo G, Ceci E, Marchetti P, et al. Occurrence of mislabeling in meat products using DNA-based assay. Journal of food science and technology. 2015;52(4):2479-84.

**Carvalho DC, et al, 2017 (Research article)**

Carvalho DC, Palhares RM, Drummond MG, Gadanho M. Food metagenomics: Next generation sequencing identifies species mixtures and mislabeling within highly processed cod products. Food control. 2017;80:183-6.

**Garcia-Vazquez E, et al, 2010 (Research article)**

Garcia-Vazquez E, Perez J, Martinez JL, PARDINas AF, Lopez B, Karaiskou N, et al. High level of mislabeling in Spanish and Greek hake markets suggests the fraudulent introduction of African species. Journal of agricultural and food chemistry. 2010;59(2):475-80.

**Staffen CF, et al, 2017 (Research article)**

Staffen CF, Staffen MD, Becker ML, Löfgren SE, Muniz YCN, de Freitas RHA, et al. DNA barcoding reveals the mislabeling of fish in a popular tourist destination in Brazil. PeerJ. 2017;5:e4006.

**Muñoz-Colmenero M, et al, 2017 (Research article)**

Muñoz-Colmenero M, Juanes F, Dopico E, Martinez JL, Garcia-Vazquez E. Economy matters: A study of mislabeling in salmon products from two regions, Alaska and Canada (Northwest of America) and Asturias (Northwest of Spain). Fisheries Research. 2017;195:180-5.

**Muñoz-Colmenero M, 2016 (Research article)**

Muñoz-Colmenero M, Blanco O, Arias V, Martinez JL, Garcia-Vazquez E. DNA authentication of fish products reveals mislabeling associated with seafood processing. Fisheries. 2016;41(3):128-38.

**Bosko SA, et al, 2018 (Research article)**

Bosko SA, Foley DM, Hellberg RS. Species substitution and country of origin mislabeling of catfish products on the US commercial market. Aquaculture. 2018;495:715-20.

**Christiansen H, et al, 2018 (Research article)**

Christiansen H, Fournier N, Hellemans B, Volckaert FA. Seafood substitution and mislabeling in Brussels' restaurants and canteens. Food Control. 2018;85:66-75.

**Galal-Khallaf A, et al, 2002 (Research article)**

Galal-Khallaf A, Ardura A, Borrell YJ, Garcia-Vazquez E. PCR-based assessment of shellfish traceability and sustainability in international Mediterranean seafood markets. Food chemistry. 2016;202:302-8.

1. **Articles on genetically modified foods**

**Swanson NL, et al, 2014(Research article)**

Swanson NL, Leu A, Abrahamson J, Wallet B. Genetically engineered crops, glyphosate and the deterioration of health in the United States of America. Journal of Organic Systems. 2014;9(2):6- 37.

**Pattron DD, 2005 (Research article)**

Pattron DD. A survey of genetically modified foods consumed, health implications and recommendations for public health food safety in Trinidad. Internet J Food Safety. 2005;7:4-14.

**Bakshi A, 2003 (Research article)**

Bakshi A. Potential adverse health effects of genetically modified crops. Journal of Toxicology and Environmental Health, Part B. 2003;6(3):211-25.

**Aris A & Leblanc S, 2011(Research article)**

Aris A, Leblanc S. Maternal and fetal exposure to pesticides associated to genetically modified foods in Eastern Townships of Quebec, Canada. Reproductive toxicology. 2011;31(4):528-33.

1. **Articles on outdated foods or foods passed their use-by dates**

**Anyanwu RC & Jukes DJ, 1991(Research article)**

Anyanwu RC, Jukes DJ. Food systems and food control in Nigeria. Food policy. 1991;16(2):112- 26.

**Burnett K, et al, 2015 (Research article)**

Burnett K, Skinner K, LeBlanc J. From Food Mail to Nutrition North Canada: reconsidering federal food subsidy programs for northern Ontario. Canadian Food Studies/La Revue canadienne des études sur l'alimentation. 2015;2(1):141-56.

**Freedman DA & Bell BA, 2009 (Research article)**

Freedman DA, Bell BA. Access to healthful foods among an urban food insecure population: perceptions versus reality. Journal of Urban Health. 2009;86(6):825-38.

**Sood M, 2014 (Field inspection)**

Sood M. The Supervision of Government on Implementation of Import of Processed Food Products in Effort of Legal Protection for Consumers. JL Pol'y & Globalization. 2014;25:72.

**Islam M, 2017 (Thesis)**

Islam M. Study on Bacteriological Quality of Street-Vended and Expired Food Items Collected from Different Areas in Dhaka City, Bangladesh: East West University; 2017.

**Kunyanga C, et al, 2011 (Research article)**

Kunyanga C, Imungi JK, Okoth MW. Diversity and characteristics of supplementary foods sold and consumed by vulnerable groups in Kenya. J Applied Biosci. 2011;45:3019-31.
